# Strategies to Manage Rice Sheath Blight: Lessons from Interactions between Rice and *Rhizoctonia solani*

**DOI:** 10.1186/s12284-021-00466-z

**Published:** 2021-02-25

**Authors:** Dayong Li, Shuai Li, Songhong Wei, Wenxian Sun

**Affiliations:** 1 College of Plant Protection, Jilin Agricultural University, 2888 Xincheng Street, 130118 Changchun, Jilin, China; 2 Department of Plant Pathology, College of Plant Protection, Shenyang Agricultural University, 110866 Shenyang, Liaoning, China; 3 Department of Plant Pathology, the Ministry of Agriculture Key Laboratory of Pest Monitoring and Green Management, China Agricultural University, 100193 Beijing, China

## Abstract

*Rhizoctonia solani* is an important phytopathogenic fungus with a wide host range and worldwide distribution. The anastomosis group AG1 IA of *R. solani* has been identified as the predominant causal agent of rice sheath blight, one of the most devastating diseases of crop plants. As a necrotrophic pathogen, *R. solani* exhibits many characteristics different from biotrophic and hemi-biotrophic pathogens during co-evolutionary interaction with host plants. Various types of secondary metabolites, carbohydrate-active enzymes, secreted proteins and effectors have been revealed to be essential pathogenicity factors in *R. solani*. Meanwhile, reactive oxygen species, phytohormone signaling, transcription factors and many other defense-associated genes have been identified to contribute to sheath blight resistance in rice. Here, we summarize the recent advances in studies on molecular interactions between rice and *R. solani*. Based on knowledge of rice-*R. solani* interactions and sheath blight resistance QTLs, multiple effective strategies have been developed to generate rice cultivars with enhanced sheath blight resistance.

## Background

Rice sheath blight (RSB) caused by the necrotrophic pathogen *Rhizoctonia solani* Kühn is considered as one of the most devastating rice diseases worldwide (Rao et al. 2020). The disease is also called “snake skin disease”, “mosaic foot stalk”, and “rotten foot stalk” because of its special disease symptoms (Molla et al. 2020; Zhang et al. 2019b). The past decades have witnessed a sharp increase in the incidence of RSB in the field largely due to the application of high dose of nitrogen fertilizers and large-scale planting of semi-dwarf high yield cultivars (Yellareddygari et al. 2014). RSB was first reported in Japan in 1910 and subsequently spread around the world, particularly in Asia, Africa and America. In China, RSB was first reported in 1934, and it has become the second most important disease in rice at present, causing a yield loss of 10 ~ 30 % every year, even up to 50 % in the rice-growing region of Yangtze river valley and South China in epidemic years (Yu et al. 2019; Zhu et al. 2019). The annual disease area in China is about 15 ~ 20 million hm^2^ (Bernardes-de-Assis et al. 2009; Shu et al. 2019).

Due to the lack of resistant germplasms in rice, progress in breeding for RSB-resistant varieties is slow. At present, chemical fungicides and cultivation practices are the major approaches to preventing and managing the disease (Singh et al. 2019; Yellareddygari et al. 2014). Advances in omics, including genomics, proteomics and transcriptomics, genetic variability of *R. solani*, quantitative trait loci (QTL) and genetic regions for RSB resistance, and integrative management methods have been well reviewed recently (Li et al. 2019a; Molla et al. 2020). This review focuses on the molecular basis of the interactions between rice and *R. solani* and the molecular strategies for controlling the disease.

## Overview of the Pathogens and Diseases

*Thanatephorus cucumeris*, the sexual stage of the causal agent of RSB, belongs to the Corticiaceae family in the Homenomycetales order of the Basidiomycetes class. The soil-borne pathogen causes diseases in many monocot and dicot plants (Anderson et al. 2017; Zhang et al. 2009). The genetically closely *R. solani* isolates are compatible and form a fused hyphal network involving fusion of cell wall, cytoplasm and nuclei, whereas the genetically distant isolates may not anastomose (Carling et al. 2002). Accordingly, *R. solani* is assigned into 14 anastomosis groups (AG1 to AG13, AGB1) with high genetic diversity based on their compatibility for hyphal fusion with known tester isolates (Carling et al. 2002; Singh et al. 2019). Based on the differences in sclerotium morphology and host plants, AG1 isolates are further divided into three subgroups, including IA, IB and IC. *R. solani* AG1 IA has been identified as the dominant anastomosis group causing RSB (Singh et al. 2019; Taheri and Tarighi 2011).

It is very difficult to find asexual spores of *R. solani* and this fungus survives in an unfavorable environment through sclerotia formed by tightly interweaving mycelia (Willetts and Bullock 1992; Sun et al. 2020b). Sclerotia are developed on infected plants late in the cropping season and are dropped into soil or water (Shu et al. 2019). Dormant sclerotia can live in the soil for several years and serve as the primary inocula for the next cropping season (Singh et al. 2019). Sclerotia can float and disperse over long distance with irrigation water and gather around rice plants on the surface of water (Yellareddygari et al. 2014; Zaeim et al. 2015). Under favorable conditions, sclerotia germinate and then the mycelia begin to grow (Feng et al. 2017; Kwon et al. 2014). *R. solani* gains entry into host tissues through multiple avenues, such as via infection cushions that are aggregates of complex hyphae or lobate appressoria that penetrate the cuticle, via stomata or wounds (Molle et al. 2013; Pooja and Babu 2017; Zhao et al. 2008). *R. solani* initially causes green-gray, water-soaked lesions on the sheath at the base of rice plants, or close to the water line in the paddy field. As the symptoms develop, the center of lesions becomes grayish white, and the edge shows dark brown. Eventually, individual lesions merge together and even cover the entire sheath. As a consequence, the sheathes become yellow and wilted, and even rot and die (Taheri and Tarighi 2011; Wang and Zheng 2018) .

## Pathogenicity Factors in ***R. solani***.

Elucidation of molecular mechanisms underlying the co-evolutionary interaction between rice and *R. solani* will facilitate developing novel strategies to manage RSB. Pathogen-secreted molecules play an important role in the pathogen-host interaction. It has been reported that the infection cushions are involved in enzymatic degradation and necrosis occurs in plants before mycelial invasion of *R. solani* (Groth and Nowick 1992; Singh et al. 2019). Besides, many defense genes in host plants are specifically down-regulated after *R. solani* infection (Rao et al. 2020; Mayo et al. 2015; Xia et al. 2017). These findings indicate that *R. solani* has evolved to secrete some substances, which actively regulate host immunity and promote virulence. Indeed, a wide arsenal of effectors, carbohydrate-active enzymes and various types of secondary metabolites secreted by *R. solani* have been identified to modulate host immunity (Costanzo et al. 2011; Wang et al. 2014; Wei et al. 2020).

### Secreted Proteins and Effectors

It is generally believed that some effector proteins secreted by hemi-biotrophic and necrotrophic pathogens can inhibit pattern-triggered immunity (PTI) at the early and transient establishment stage of infection, while others induce cell death at the late necrotrophic stage of infection and ultimately contribute to pathogen virulence (Li et al. 2019c; Wei et al. 2020). Through comparative genomics and secretome analyses, 985 secreted proteins and 103 small cysteine-rich effector candidates were predicted in *R. solani* AG1 IA genome (Zheng et al. 2013). The effectors AG1IA_09161, AG1IA_05310 and AG1IA_07795 containing glycosyltransferase GT family 2, cytochrome C oxidase assembly protein CtaG/cox11 and peptidase inhibitor I9 domains, respectively, have been identified to induce cell death in rice and maize plants. Eighteen putative effectors were subsequently identified and categorized into three classes of verified effectors through the hierarchical clustering method (Zheng et al. 2013). Based on transcriptome analyses, hundreds of putative secreted protein-encoding genes and dozens of candidate effector genes are up-regulated during the infection of *R. solani* AG1 IA (Ghosh et al. 2018; Xia et al. 2017). Among them, a nucleoside diphosphate-linked moiety X (Nudix) domain-containing protein (AG1IA_02392), NACHT domain-containing protein (AG1IA_06487) and BTB domain-containing protein (AG1IA_03906) are preferentially expressed during *R. solani* infection of the susceptible rice cultivars (Rao et al. 2020). Although Nudix effectors are widely distributed in plant pathogens and probably play essential roles in pathogenesis, very few have been characterized (Dong et al. 2016). Therefore, it is important to reveal the role of AG1IA_02392 in *R. solani* pathogenicity (Rao et al. 2020). Recently, a putative lipase effector AGLIP1 has been demonstrated to induce cell death in *Nicotiana benthamiana* and rice protoplasts. Interestingly, ectopic expression of AGLIP1 suppresses basal defenses and promotes bacterial multiplication in Arabidopsis ( Li et al. 2019c). Besides, a highly conserved fungal effector RsIA_NP8 in *R. solani* anastomosis group AG1 is localized predominantly in the chloroplasts and triggers non-host cell death when transiently expressed in *N. benthamiana* (Wei et al. 2020).

Chitin, an unbranched homopolymer of 1, 4-β linked N-acetyl-D-glucosamine (GlcNAc), is the second most abundant polysaccharide on earth and is the major component of fungal cell walls. As a major pathogen-associated molecular pattern (PAMP) in rice, chitin is recognized by the pattern recognition receptor (PRR) CEBiP and coreceptor OsCERK1 on plant cell membrane, and thus initiating immune responses (Gong et al. 2020; Sanchez-Vallet et al. 2020; Shimizu et al. 2010). For successful infection, fungal pathogens have evolved to secrete certain effector proteins to interfere with PAMP recognition by PRRs. In the soil-borne *Verticillium* and *Fusarium* fungal pathogens, secretory polysaccharide deacetylase (PDA1) proteins deacetylate chitin oligomers into ligand-inactive chitosan, and thereby preventing chitin-triggered immunity and promoting virulence (Gao et al. 2019). Gene expression profiling reveals that a chitin deacetylase-encoding gene (CL8196Contig1) is significantly up-regulated during *R. solani* infection (Ghosh et al. 2018). Therefore, it is of interest to investigate whether *R. solani* exploits the same strategy to inhibit chitin-triggered host immunity. Besides, *R. solani* encodes the carbohydrate binding module 14 (CBM14) family of putative chitin-binding proteins similar to *Cladosporum fulvum* Avr4 and Ecp6, which suppress chitin-triggered immunity through blocking the release of chitin from fungal cell walls by plant chitinases and competing with CEBiP for binding of chitin oligosaccharides, respectively (Anderson et al. 2017; de Jonge et al. 2010; van den Burg et al. 2006). Additionally, the genes encoding xylanase and inhibitor I9 domain-containing proteins are transcriptionally up-regulated during *R. solani* infection. Both proteins can induce plant cell death when transiently expressed in *N. benthamiana* (Anderson et al. 2017). Collectively, the effectors in *R. solani* might function as important pathogenicity factors. Elegant studies from *Sclerotinia sclerotiorum* also substantiate the essentiality of effector proteins in virulence and pathogenicity of the necrotrophic pathogens (Liang and Rollins 2018). Next, it is interesting to identify the effectors that inhibit pattern-triggered immunity to establish initial colonization and also the effectors that induce cell death and contribute to necrotrophic stage.

### Secondary Metabolites

*R.solani* secretes a variety of secondary metabolites, including host-selective toxins and biologically active molecules. These factors contribute to pathogen virulence through breaking host physical barriers and interfering with normal physiological functions and host defenses (Brooks 2007; Costanzo et al. 2011; Howlett 2006). The host-specific toxin in *R. solani* has been partially purified and identified as a carbohydrate consisting of mannose, N-acetylglucosamine, glucose and N-acetylgalactosamine. Highly virulent *R. solani* isolates produce more host-specific toxin than weakly virulent isolates (Chen et al. 2009; Vidhyasekaran et al. 1997; Yang et al. 2011).

Other biologically active molecules produced by *R. solani* include oxalic acid (OA), 3-methylthiopropionic acid (MTPA), phenylacetic acid (PAA) and its derivatives (Brooks 2007; Hu et al. 2018; Vidhyasekaran et al. 1997; Yang et al. 2014). OA produced by necrotrophic pathogens is an essential virulence factor for successful infection. The highly virulent isolates of *R. solani* have been identified to produce more OA than weakly virulent isolates (Nagarajkumara et al. 2005). During *R. solani* infection, OA inhibits the synthesis of various phenolic substances and acts synergistically with polygalacturonases (PGs) to cause pH instability and cell wall degradation (Liang et al. 2018; Qi et al. 2017; Rollins and Dickman, 2001). Besides, OA triggers programmed cell death in host plants, and thus promoting infection (Cessna et al. 2000; Heller et al.^ 2013^; Kabbage et al. 2013). PAA treatment, similar to *R. solani* infection, causes severe disease symptoms on maize sheath (Cook et al. 2016; Hu et al. 2018). Besides, PAA treatment and *R. solani* infection have similar effects on the synthesis of secondary metabolites, including traumatin, phytosphingosine, vitexin 2’’ O-beta-D-glucoside, rutin and 2,4-dihydroxy-2H-1,4-benzoxazin-3(4H)-one (DIBOA)-glucoside, while inhibit the generation of the jasmonic acid (JA) precursors 3-oxo-2-(2’-pentenyl)-cyclopentane-1-octanoic acid (OPC-8:0) and 12-oxo-phytodienoic acid (OPDA) (Ahmad et al. 2011; Hu et al. 2018; Yang et al. 2016). The PAA metabolism pathway has been well identified and the phytotoxin inhibits seed germination and chlorophyll synthesis and functions as a virulence factor in the potato pathogen AG3 (Chen et al. 2009; Lakshman et al. 2006). Unexpectedly, it fails to directly isolate or detect PAA in *R. solani* AG1 IA. The amount of PAA and its derivatives detected in the culture medium of the AG1 IA isolates is also significantly lower than that from AG3 and AG4, indicating PAA might not be the primary toxin for AG1 IA (Bartz et al. 2012; Kankam et al. 2018). It has also been demonstrated that MTPA is essential for mycelial production and promotes disease progression (Kankam et al. 2018).

Furthermore, the cytochrome P450s (CYPs)-encoding genes involving in the biosynthesis of primary and secondary metabolites including phytotoxins are differentially regulated during *R. solani* infection and sclerotial development, implying that the *CYPs* and *CYP*-associated genes contribute to *R. solani* pathogenicity (Shu et al. 2019; Xia et al. 2017). These findings further highlight the essentiality of secondary metabolites in *R. solani* pathogenicity (Ghosh et al. 2019; Keller et al. 2005; Vidhyasekaran et al. 1997; Yang et al. 2014).

### Carbohydrate‐active Enzymes

For successful invasion, plant fungal pathogens generally secrete various types of carbohydrate-active enzymes (CAZymes), particularly including cell wall-degrading enzymes (CWDEs) (Molla et al. 2020; Rao et al. 2020). CAZymes mediate the degradation of cellulose, hemicellulose and pectin in host cell walls and break down the physical barrier of plant immune system, and thus enhancing pathogen virulence (Zheng et al. 2013; Xia et al. 2017). CWDEs in *R. solani* are categorized into seven major families including PG, endo-β- 1,4-glucanase, polymethylgalacturonase, pectin methylesterase, filter paper enzyme, polymethylgalacturonase trans-eliminas and pectin methyltrans-eliminase (Silva et al. 2018; Xue et al. 2018; Rathinam et al. 2020). As a group of hydrolytic enzymes, PGs act on polygalacturonic acid, a key component of plant cell wall, by cleaving the α-(1,4)-glycosidic bonds, and thus causing hydrolysis of pectate. Pathogens of rice, soybean, maize and other crops have evolved multiple PGs to maximize their offensive potential (Chen et al. 2006; Gawade et al. 2017; Zhou et al. 2016). *R. solani* AG1 IA selectively secretes various pectinases according to the cell wall characteristics of different host species (Zheng et al. 2013). RNAseq analysis revealed that 30 genes encoding pectin-degrading enzymes in *R. solani*, especially *PG_04727*, *PG_01811*, *PG_06500*, *PE_09779* and *AG1IA_01129*, were greatly induced during infection, indicating that these pectin-degrading enzymes are likely involved in *R. solani* AG1 IA infection of rice (Rao et al. 2020). Many CAZyme genes encoding pectin lyases, cellobiose dehydrogenase and β-glucanases are also up-regulated at different levels during *R. solani* infection of the hosts (Ghosh et al. 2018; Rao et al. 2020). Besides, endo-1,4-β-xylanase A breaks down hemicellulose in plant cell wall through decomposing linear β-1,4-xylan into xylose to facilitate pathogen penetration. The xylanase shows increased expression in highly susceptible rice plants after *R. solani* infection (Prathi et al. 2018).

In addition, other transcriptome analyses suggest that a variety of glycosyltransferases (GTs), glycoside hydrolases (GHs), polysaccharide lyases (PLs), carbohydrate esterases (CEs) and non-catalytic CBMs are the important CAZymes involving in the degradation of host cell walls and in *R. solani* virulence (Ghosh et al. 2018; Xia et al. 2017; Zheng et al. 2013). Sixteen GH-, GT-, and PL-encoding genes exhibit significantly higher expression levels in the rice susceptible cultivars compared with the resistant cultivars after *R. solani* inoculation, which might explain the phenotype of higher degree of necrosis in susceptible cultivars (Rao et al. 2020). Notably, much more CAZymes are up-regulated in *R. solani* during the necrotrophic phase compared with the colonization stage, implying that CAZymes in necrotrophic pathogens play more important roles in virulence than those in biotrophic pathogens (Blanco-Ulate et al. 2014; Ghosh et al. 2018; Zhao et al. 2013). This statement can stand close scrutiny since biotrophic pathogens generally avoid damaging the cells to acquire nutrients and restrict the cell wall degradation, which products are often recognized as damage-associated molecular patterns and thus triggering plant defenses.

## Countermeasures from the Host

To counteract the effects from pathogenicity factors in plant pathogens, plants develop multiple layers of defenses against pathogen attacks. During pathogen infection, PAMPs are recognized by plant PRRs, and thus triggering PTI. As the first layer of plant defense, PTI responses include the activation of defense gene expression and mitogen-activated protein kinase (MAPK) cascades, reactive oxygen species (ROS) burst, accumulation of secondary metabolites and defense-related phytohormone signaling pathways (Bigeard et al. 2015; Yu et al. 2017; Zipfel 2014). On the other hand, intercellular immune receptors (R proteins) in host plants recognize certain pathogen effectors and therefore cause hypersensitive responses, which are rapid and robust defense responses called effector-triggered immunity (Jones and Dangl 2006; Peng et al. 2018). The two branches of plant immunity are perfectly coordinated and might cross-talk with each other to form a barrier against external biological invasion.

### Reactive Oxygen Species

In general, ROS has a dual function in regulating plant defenses. ROS can function as a signal molecule initiating defense responses against fungal pathogens. In response to *R. solani* challenge, plants generate ROS and initiate various defense responses, such as phytoalexin biosynthesis, activation of defense-related genes and callose deposition in cell walls (Gechev et al. 2006). H_2_O_2_ activates phenylalanine ammonia lyase (PAL)-mediated biosynthesis of phenolic compounds including lignin in the shikimate pathway, and thereby improves plant antioxidant capacity and thickens cell walls (Molla et al. 2013). Particularly in the early stage of infection, plants prevent pathogen infection by enhancing cell wall lignification and producing a variety of secondary metabolites. Comprehensive KEGG enrichment analyses also indicate that phenylpropanoid and phenylalanine metabolism plays an important role in preventing infection in RSB resistant varieties (Kwon et al. 2014; Shi et al. 2020; Zhang et al. 2017).

Interestingly, the rice resistant line exhibits a significant less ROS accumulation compared with the susceptible line at 48 h post inoculation of *R. solani* (Oreiro et al. 2020). In this case, pathogen attack may stimulate excessive ROS accumulation in the susceptible plants and thereby inducing cell death, which is conducive to the growth and colonization of necrotrophic fungi (Heller and Tudzynski 2011). Therefore, ROS might function as a signal for *R. solani* to switch from the establishment to the necrotrophic stage (Noctor et al. 2018; Oreiro et al. 2020). Besides, exogenous and endogenous ROS has a promoting effect on the formation and development of *R. solani* sclerotia, while trehalose, a ROS-scavenger, functions as an inhibitor to sclerotial development, suggesting that trehalose might serve as a new antioxidant fungicide to inhibit sclerotial differentiation in *R. solani* AG-1 IA ( Wang et al. 2018).

To effectively balance the role of ROS during the interaction between rice and *R. solani*, many smart strategies have evolved to regulate the ROS level to reduce damage and activate defense in rice. A quantitative trait locus *qLN11*^*28*^ enriched in defense-related genes is involved in the activation of the genes related to the ROS-redox pathway and alleviates ROS accumulation in rice cells, and thus delaying *R. solani* colonization (Oreiro et al. 2020). The catalase OsCATC and chloroplast glutathione peroxidase involving in scavenging ROS are found in hub gene network in rice and might play an important role in maintaining normal ROS level in rice after *R. solani* infection (Alam and Ghosh 2018). In addition, previous studies revealed that various peroxidases can function to maintain the balance of H_2_O_2_ and play an important role in cell wall regeneration and thickening (Bindschedler et al. 2006; Marjamaa et al. 2009).

### Phytohormones and Transcription Factors

Phytohormone signaling pathways are required for plant defenses against various pathogens. Typically, SA confers resistance against biotrophic and hemibiotrophic pathogens, whereas JA and ethylene synergistically cooperate to activate defenses against necrotrophs (Singh et al. 2018). The ethylene insensitive mutant sickle (*skl*) is highly susceptible to *R. solani* AG8, while the transgenic plants overexpressing ethylene response factors (ERFs) or the ethylene biosynthesis enzyme ACS2 exhibit resistance to *R. solani* (Anderson et al. 2010; Anderson et al. 2018; Helliwell et al. 2013). Ethylene-insensitive protein 2 (EIN2) is significantly up-regulated in the resistant and susceptible rice varieties during *R. solani* infection (Shi et al. 2020). These findings support that ethylene signaling positively regulates rice resistance to RSB. An elegant study has revealed that siR109944, a type of tourist-miniature inverted-repeat transposable element-derived small interfering RNA, suppresses rice immunity to sheath blight by affecting auxin homeostasis. The small RNA targets *F-Box domain- and LRR-containing protein 55* (*FBL55*) encoding transport inhibitor response 1 (TIR1)-like protein. The *siR109944*-overexpressing rice plants have a significantly enhanced susceptibility to *R. solani*, while FBL55-overexpression causes the transgenic plants to be more resistant to RSB (Qiao et al. 2020). Additionally, multiple indeterminate domain (IDD) proteins including LPA1 (Loose Plant Architecture 1), IDD3 and IDD13 form a transcription factor complex and regulate expression of the auxin efflux carrier gene *PIN-FORMED 1a* (*PIN1a*) in rice through binding to its promoter (Sun et al. 2019; Sun et al. 2020a). Interestingly, LPA1 and IDD13 positively regulate *PIN1a* expression and RSB resistance, while IDD3 is a negative regulator in *PIN1a* expression and RSB resistance (Sun et al. 2019; Sun et al. 2020a). The G-protein γ subunit DEP1 (dense and erect panicle 1) interacts with LPA1 and suppresses DNA binding ability of LPA1 and thereby inhibiting expression of *PIN1a*. Consistently, mutation of G-protein γ subunit DEP1 can promote rice resistance to RSB (Liu et al. 2020; Sun et al. 2019; Sun et al. 2020a). Furthermore, auxin application enhances rice resistance to sheath blight (Qiao et al. 2020; Sun et al. 2019). These results indicate that auxin signaling positively regulates RSB resistance and that application of auxin analogues might effectively protect field crops against the disease.

The transcription factors including NAC, WRKY, and basic leucine zipper (bZIP) family members differentially regulate the expression of various defense genes and are involved in modulating resistance to *R. solani* (Olsen et al. 2005; Zhang et al. 2018). NAC transcription factors are involved in the regulation of signal transduction pathways mediated by salicylic acid (SA), JA, ethylene and abscisic acid (Zhao et al. 2020). The *osnac4* mutant shows reduced necrosis symptom after pathogen infection, while *OsNAC4* overexpression promotes *R. solani* infection in rice, indicating that OsNAC4 negatively regulates sheath blight resistance in rice (Kaneda et al. 2009). By contrast, the NAC transcription factor XNDL positively modulates RSB resistance through activating the ethylene signaling pathway (Wang 2019). Multiple WRKY family members are also important components of plant defense against pathogen infection (Gallou et al. 2012; Shi et al. 2020; Zheng et al. 2013). OsWRKY30 and OsWRKY80 are positive regulators in rice resistance against sheath blight (Peng et al. 2012; Peng et al. 2016). Silencing of *OsWRKY80* in rice remarkably reduces resistance to *R. solani* (Peng et al., 2016). *OsWRKY*80 expression is induced by *R. solani* infection, exogenous JA and ethylene, but not by SA. OsWRKY80 elevates *OsWRKY*4 expression through specifically binding to the promoter of *OsWRKY*4 and both of transcription factors function synergistically to increase rice defenses against *R. solani* infection (Peng et al. 2016; Wang et al. 2015a). Over-expression of *WRKY*13 in rice results in significantly increased expression of *WRKY*12, *TIFY*9 and *PR*2 and enhances resistance to *R. solani* (Jimmy and Babu 2019). *WRKY*33 and *WRKY*70 are significantly induced in the rice susceptible cultivars in comparison with the resistant cultivars after *R. solani* infection. Various types of transcription factors are often involved in regulating different phytohormone signaling, and thereby modulating rice defenses against sheath blight. WRKY70 acts as an activator of SA-induced genes and a repressor of JA-responsive genes (Shi et al. 2020; Zhang et al. 2017). The transcription factor RAVL1 negatively regulates rice resistance to *R. solani* through activating the expression of brassinosteroid-related genes (Helliwell et al. 2011; Yuan et al. 2018). Paradoxically, RAVL1 also activates ethylene-mediated signaling, which positively regulates rice resistance against sheath blight (Yuan et al. 2018). These findings indicate that rice plants have evolved distinct strategies to defend against various pathogens, which might be mediated by different transcription factors.

### Other Signal Molecules

As an important secondary messenger, Ca^2+^ regulates plant physiology and metabolism and plays an important role in plant disease resistance (Pusztahelyi et al. 2015). After *R. solani* infection, most of the genes involving in the calcium signaling pathway in rice are down-regulated at 12 h but increased at 24 h, which is more significant in resistant varieties compared with susceptible varieties, implying that these genes are involved in regulating sheath blight resistance (Zhang et al. 2017). The calcium-binding proteins OsBON1 and OsBON3 have been identified as negative regulators of broad-spectrum disease resistance to both bacterial and fungal pathogens including *R. solani*, which regulate the balance between immunity and agronomic traits in rice (Yin et al. 2018).

To combat OA action, host plants have evolved to effectively decompose oxalate by synthesizing and secreting oxalate oxidases (Karmakar et al. 2015; Molla et al. 2013). Bacteria and fungi are also able to produce oxalate decarboxylases (ODCs) to inhibit OA accumulation (Liang et al. 2015; Qi et al. 2017). During *R. solani* colonization of host plants, several *ODC* genes were significantly up-regulated (Ghosh et al. 2019; Liang et al. 2015). The *ODC* genes are essential for the formation of appressorium in *S. sclerotiorum*, another necrotrophic fungal pathogen, probably because ODCs function in OA detoxification and pH homeostasis or contribute to catabolic energy generation (Liang et al. 2015).

As a countermeasure to fungal PGs, plant polygalacturonase-inhibiting proteins (PGIPs) specifically recognize and bind to fungal PGs, and thereby inhibit the cell wall degradation activity and prevent fungal infection (Rathinam et al. 2020). The interaction of PGs and PGIPs results in the production of oligogalacturonides, which are important signals to induce *PGIP* gene expression and plant defense responses (Davidsson et al. 2017). The expression of the *PGIP* family genes is also significantly induced by *R. solani* infection, wounding, SA and JA (Feng et al. 2016; Zhu et al. 2019). It has been demonstrated that multiple PGIPs from different plant species except OsPGIP2 confer a wide spectrum of inhibitory activities to PGs and play an essential role in resistance against various fungal pathogens including *R. solani* (Wang et al. 2015b; Zhu et al. 2019).

### Lessons from the Rice - ***R. solani*** Interaction

No cultivar with complete resistance has been identified after screening of thousands of rice cultivars from various rice growing regions (Li et al. 2019a; Molla et al. 2020; Shi et al. 2020). Due to the lack of resistant germplasms to RSB, successes in conventional breeding programs for RSB resistant varieties are very limited. By contrast, many pathogenicity factors in *R. solani* and the underlying mechanisms have been revealed in the molecular interaction between rice and *R. solani* (Table 1). Besides, many defense-related genes have been also identified to be associated with sheath blight resistance in rice (Table 2). The knowledge provides a solid foundation to create RSB resistant germplasms through precisely manipulating the expression of target genes via transgenic technology, gene editing and RNA interference (Table 1).

**Table 1:** The effective strategies to generate rice cultivars with enhanced sheath blight resistance are developed based on the findings in molecular interactions between rice and *R. solani*

Essential pathogenicity factors in *R. solani*		The strategies and genes/proteins used to enhance rice sheath blight resistance		Promoters/Transformation methods	References
Name	Roles during infection	Name	Functions		
Oxalic acid	Oxalic acid (OA) is secreted and accumulated early in the pathogen–plant interaction, and is involved in plant cell wall degradation.	*OsOXO4*	The oxalate oxidase detoxifies OA and generates CO_2_ and H_2_O_2_, which triggers defense responses in plants.	A green tissue-specific promoter-cassette, the biolistic method	Molla et al. (2013)
		*OxDC* (*Bacisubin*)	The oxalate decarboxylase (OxDC) from *Bacillus subtilis* catalyzes the production of formic acid and CO_2_ from OA, which reduces the accumulation of oxalic acid.	The CaMV35S promoter, *Agrobacterium tumefaciens*-mediated transformation	Qi et al. (2017)
*RPMK*1-1 and *RPMK*1-2	*PMK*1 homologs are essential for the formation of appressorium, the fungal infection srtructures, and invasive growth inside the plant.		Host-derived RNA interference is used to silence *RPMK*1-1 and *RPMK*1-2 in *R. solani.*	Embryogenic calli as a target tissue using a biolistic particle gun	Tiwari et al. (2017)
*AG1IA_04727*	The gene encodes polygalacturonase.		Host-derived RNA interference (Silencing)	Constitutive maize ubiquitin promoter, *A. tumefaciens*-mediated transformation	Rao et al. (2019)
Polygalacturonase	Polygalacturonases (PGs) secreted by *R. solani* degrade pectin, which is a major plant cell wall component.	*OsPGIP1*	The polygalacturonase inhibiting proteins (PGIPs) can specifically recognize PGs to prevent fungal infection through inhibiting their cell wall degradation activity.	The CaMV35S promoter, *A. tumefaciens* -mediated transformation	Wang et al. (2015b)
		*OsPGIP2*^*L233F*^		The maize ubiquitin-1 promoter, *A. tumefaciens*-mediated transformation	Chen et al. (2019)
		*ZmPGIP3*			Zhu et al. (2019)
PAA	A biologically active toxin molecule produced by *R. solani*	Glyoxalase	The glyoxalase detoxifies the cytotoxic metabolite methylglyoxal generated in response to biotic and abiotic stresses in plants.	The CaMV35S promoter, *A. tumefaciens* -mediated transformation	Gupta et al. (2017)
α-1,3-glucan	masking chitin to evade PRR recognition, and maintaining the infection-specific hyphal structure	α-1,3- glucanase	A bacterial α-1,3-glucanase is able to remove α-1,3-glucan on the fungal surfaces.	The CaMV35S promoter, *A. tumefaciens* -mediated transformation	Fujikawa et al. (2012)

**Table 2:** The plant defense-related genes that have been manipulated to enhance sheath blight resistance in rice

		Promoters/Transformation methods	Resistance phenotype	References
Name	Functions			
*OsCHI11*	The chitinases are chitin-degrading enzymes that hydrolyze the β-(1, 4) linkages of chitin.	The maize ubiquitin promoter or CaMV35S promoter, *A. tumefaciens*-mediated transformation or protoplast transformation	The transgenic plants overexpressing OsCHI11 exhibit enhanced resistance to sheath blight.	Lin et al. (1995) Baisakh et al. (2001) Kumar et al. (2003) Sridevi et al. (2003) Sridevi et al. (2008) Sripriya et al. (2008)
*OsRC7*	The chitinase gene (*OsRC7*) encodes a class I chitinase (PR3 family) in rice.	The CaMV35S promoter, the biolistic method and protoplast transformation	The transgenic plants overexpressing the chitinase show different levels of enhanced resistance to *R. solani.*	Datta et al. (2001)
*Os11g47510*	A novel chitinase gene from *indica* rice	The CaMV35S promoter, the biolistic method	The transgenic plants show good correlation between transgene expression and the level of sheath blight resistance.	Richa et al. (2017)
*Ace*-*AMP1*	Ace-AMP1, a cysteine rich antimicrobial peptide from *Allium cepa*, is homologous to plant ns-LTP and shows strong antimicrobial activity.	An inducible rice phenylalanine ammonia-lyase (*PAL*) promoter or a constitutive maize ubiquitin promoter, *A. tumefaciens*-mediated transformation	The transgenic plants exhibit increased resistance to blast, sheath blight, and bacterial leaf blight without agronomic trait penalty.	Patkar and Chattoo (2006)
*Dm*-*AMP1*	Dm-AMP1, an antifungal plant defensin from *Dahlia merckii*, directly inhibits the pathogen.	The maize ubiquitin promoter, *A. tumefaciens*-mediated transformation	The transgenic rice plants expressing Dm-AMP1 have the potential to provide broad-spectrum disease resistance in rice.	Jha et al. (2009)
*Rs*-*AFP2*	Rs-AFP2, an antifungal plant defensin from *Raphanus sativus*, directly inhibits the pathogen.	The maize ubiquitin promoter, *A. tumefaciens*-mediated transformation	The transgenic rice plants expressing Rs-AFP2 show enhanced resistance to *M. oryzae* and *R. solani*.	Jha and Chattoo (2010)
*OsWRKY30*	WRKY30 activates expression of JA synthesis-related genes *LOX*, *AOS2* and pathogenesis-related *PR*3 and *PR*10 genes	The maize ubiquitin promoter, *A. tumefaciens*-mediated transformation	The transgenic lines overexpressing *WRKY30* have an enhanced resistance to *R. solani*.	Peng et al. (2012)
*OsWRKY80*	OsWRKY80 activates OsWRKY4 expression, and subsequently activates JA/ET-dependent defense responses.	The maize ubiquitin promoter, *A. tumefaciens*-mediated transformation	Overexpression of *OsWRKY80* in rice plants significantly enhances disease resistance to *R. solani*, while silencing of *OsWRKY80* compromises disease resistance to *R. solani*.	Peng et al. (2016)
*OsACS2*	OsACS2,1-aminocyclopropane-1-carboxylic acid synthase, is a key enzyme of ET biosynthesis.	The pathogen-inducible *PBZ1* promoter, *A. tumefaciens*-mediated transformation	The OsACS2-overexpression lines show significantly increased levels of endogenous ET and defence gene expression, increased resistance to *R. solani* and *M. oryzae*, with little or no influence on seed production.	Helliwell et al. (2013)
*AtNPR1*	Nonexpresser of pathogenesis-related gene 1 (NPR1) in Arabidopsis as the master regulator of salicylic acid-mediated signaling	The CaMV35S promoter, *A. tumefaciens*-mediated transformation	Green tissue-specific expression of *AtNPR1* in rice confers resistance to sheath blight, with no concomitant abnormalities in plant growth and yield parameters.	Molla et al. (2016)
*BjNPR1*	*Brassica juncea* nonexpresser of pathogenesis-related gene 1 (BjNPR1) causes redox-regulation in mustard akin to that of AtNPR1 in *Arabidopsis.*	The CaMV35S promoter, *A. tumefaciens*-mediated transformation	The BjNPR1-expressing rice lines display enhanced resistance to rice blast, sheath blight and bacterial leaf blight diseases, and improved agronomic traits.	Sadumpati et al.(2013)
*OsOSM1*	Osmotins belong to thaumatin-like proteins of PR5 family and are involved in the permeability stress and defense response in plants	The maize ubiquitin promoter, *A. tumefaciens*-mediated transformation	An appropriately elevated level of OsOSM1 in transgenic rice enhances RSB resistance without affecting rice development or grain yield.	Xue et al. (2016)
*OsOXO4* and *OsCHI11*	As mentioned above	The green tissue-specific rice *D54O-544* promoter and maize *PEPC* promoter, *A. tumefaciens*-mediated transformation	Simultaneous overexpression of the defense-related genes *OsCHI11* and *OsOXO4* in rice leads to significant resistance against *R. solani* without distressing any agronomically important traits.	Karmakar et al.(2015)
*AtNPR1* and *OsCHI11*	As mentioned above	The green tissue-specific rice *D54O-544* promoter and maize *PEPC* promoter, *A. tumefaciens*-mediated transformation	The transgenic rice plants with simultaneous overexpression of *AtNPR1* and *OsCHI11* show a significant upregulation of defense-related genes, *PR* genes, and antioxidant marker genes, and are more resistant to sheath blight as compared to the single transgene.	Karmakar et al.(2017)
*OsMAPK20-5*	Mitogen-activated protein kinases (MAPKs) play important roles in plant responses to biotic stresses.	Silencing of *OsMAPK20-5* by inserting an inverted-repeat orientation (irMAPK20-5) *A. tumefaciens*-mediated transformation	Silencing of *OsMAPK20-5* causes rice plants more susceptibility to *Cnaphalocrocis medinalis* and *Magnaporthe grisea*, but enhances sheath blight resistance.	Liu et al. (2019)
*OsGSTU5*	OsGSTU5 is a tau class of glutathione-S-transferase in rice, an important defense-associated protein that confers resistance against several abiotic and biotic stresses.	Via the expression vector pIRS154, *A. tumefaciens*-mediated transformation	The *OsGSTU5*-overexpressing rice lines are more tolerant, while the knockdown lines are more prone to *Rhizoctonia* infection.	Tiwari et al. (2020)
*OsASR2*	OsASR2 (abscisic acid, stress and ripening 2) regulates the expression of a defense-related gene, *Os2H16*, by targeting the GT-1 *cis*-element.	Maize Ubiquitin 1 promoter, *A. tumefaciens*-mediated transformation	Overexpression of *OsASR2* enhances the resistance against *Rhizoctonia solani* and tolerance to drought in rice.	Li et al. (2018)
*LPA1*	LPA1 belongs to an INDETERMINATE DOMAIN protein family and regulates sheath blight resistance, tiller and leaf angle by activating auxin signaling.	Maize Ubiquitin 1 promoter, *A. tumefaciens*-mediated transformation	Overexpression of *LPA1* and *IDD13* enhances rice defenses against sheath blight via the activation of *PIN1a* in rice.	Sun et al. (2019) Sun et al. (2020a, 2020b)
*DEP1*	DEP1, a G-protein γ subunit, is a novel interactor of LPA1 and controls dense and erect panicle of rice.	The knock-out mutant *dep1* (PFG_3A-02648) was generated with T-DNA insertion.	*DEP1* knock-out increases planting density and resistance to sheath blight disease in rice.	Liu et al. (2020)

### Silencing Essential Pathogenicity Genes via RNA Interference in the Fight Against ***R. solani***

Cross-kingdom trafficking of small RNAs (sRNAs) between hosts and pathogens opens an avenue to develop disease resistant transgenic plants producing sRNAs and double-stranded RNAs, which can silence fungal pathogenicity genes (Huang et al. 2019; Wang et al. 2016). The host delivered RNA interference (HD-RNAi) technology has been developed to silence two PATHOGENICITY MAP KINASE 1 (*PMK*1) homologues, *RPMK*1-1 and *RPMK*1-2 in *R. solani*. The transgenic rice plants show an increased resistance to RSB (Tiwari et al. 2017). Besides, silencing of the key pathogenicity gene *AG1IA_04727* encoding polygalacturonase via HD-RNAi significantly enhances rice resistance to *R. solani* (Rao et al. 2019). These results indicate that the targeting of key pathogenicity genes in *R. solani* by HD-RNAi is a novel and promising strategy for durable control of RSB.

### Targeting Essential Pathogenicity Factors in ***R. solani*** via Transgenic Technology

The attempts at inhibiting the PG activity via PGIP overexpression are also successful in suppressing *R. solani* infection. Overexpression of OsPGIP1 significantly improves rice resistance to RSB (Chen et al. 2016; Rathinam et al. 2020; Wang et al. 2015b). Although OsPGIP2, a homolog of OsPGIP1, has no inhibitory activity to PGs, the mutant protein OsPGIP2^L233F^ has been identified to have the PG inhibition activity. Overexpression of OsPGIP2^L233F^ confers rice resistance to *R. solani* (Chen et al. 2019). Furthermore, the transgenic rice plants constitutively expressing ZmPGIP3 exhibit significantly elevated expression of some rice *PGIP* genes and enhanced resistance to sheath blight compared with the wild-type plants (Zhu et al. 2019). Importantly, these transgenic plants do not show any detrimental phenotypic or agronomic effect. The findings indicate that genome editing and natural allele mining of plant *PGIP* genes provide important strategies to improve RSB resistance in rice.

Since OA is an essential pathogenicity factor for necrotrophic pathogens, expression of OA-detoxifying enzymes in host plants leads to enhanced resistance against necrotrophic pathogens including *R. solani* (Nagarajkumara et al. 2005; Liang and Rollins 2018). Overexpression of the rice oxalate oxidase 4 gene (*OsOXO4*) and simultaneous overexpression of *OsOXO4* and the chitinase gene *OsCHI11* driven by green tissue-specific promoters both significantly confer enhanced and durable resistance to sheath blight (Karmakar et al. 2015; Molla et al. 2013). Expression of the oxalate decarboxylase Bacisubin, an oxalate-degrading enzyme from *Bacillus subtilis*, also enhances resistance to RSB and fungal blast diseases (Qi et al. 2017). In the transgenic plants expressing oxalate oxidases and ODCs, OA released by *R. solani* is degraded by these OA-detoxifying enzymes to generate H_2_O_2_. Hydrogen peroxide plays a key role in activating defense responses, such as phytoalexin biosynthetic pathways, hypersensitive response, systemic acquired resistance, and subsequently induces the expression of *PR* genes TS and RC24 genes (Molla et al. 2013; Qi et al. 2017).

As another essential virulence factor in *R. solani*, the phytotoxin PAA induces the production of the cytotoxic metabolite methylglyoxal (MG) in rice as a common consequence of many abiotic and biotic stresses. The transgenic rice plants overexpressing glyoxalase for MG detoxification have been demonstrated to have much less accumulation of MG and enhanced resistance towards damage caused by PAA. The finding provides another transgenic technology to develop RSB resistant rice plants (Gupta et al. 2017). The mechanism of observed tolerance of the glyoxalase-overexpressing plants towards diverse abiotic and biotic stresses involves enhanced detoxification and reduced oxidative damage, leading to better protection of chloroplast and mitochondrial ultrastructure and maintained photosynthetic efficiency under stress conditions (Gupta et al. 2017).

Interestingly, α-1,3-glucan produced by *R. solani* masks chitin to evade PRR recognition, thus promoting pathogen infection. Besides, α-1,3-glucan is essential for the maintenance of the infection-specific hyphal structure (Fujikawa et al. 2012). These findings provide a new virulence target for developing rice plants against *R. solani* infection. Actually, the transgenic rice plants expressing and secreting bacterial α-1,3-glucanase show strong resistance not only to *R. solani* but also to the phylogenetically distant ascomycete *Cochlioborus miyabeanus* and *Magnaporthe oryzae* (Fujikawa et al. 2012).

### Enhancing Sheath Blight Resistance by Manipulating Expression of Plant Defense-associated Genes

Although no complete resistance gene for sheath blight has been identified in rice, many successful attempts have been performed to develop resistant rice lines by expressing defense-associated genes. Non expresser of pathogenesis-related genes 1 (NPR1) was first identified in Arabidopsis to be a master regulator of systemic acquired resistance, which confers broad-spectrum resistance to various pathogens (Fu and Dong 2013). Tissue-specific expression of Arabidopsis *NPR1* gene in rice enhances sheath blight resistance without phenotypic and agronomic costs (Molla et al. 2016). The transgenic *indica* rice lines expressing *Brassica juncea* NPR1 also exhibit enhanced resistance to *R. solani* (Sadumpati et al. 2013). Overexpression of pathogenesis-related genes such as *PR*3 and *PR*5 results in enhanced resistance to *R. solani*, manifested by reduced disease lesion sizes in the transgenic rice plants as compared with the wild-type rice plants (Datta et al. 1999; Datta et al. 2001; Datta et al. 2002). In addition, over-expression of OsGSTU5, a tau class glutathione-S-transferase, in rice effectively increases the activities of superoxide dismutase and peroxidase, and thereby reduces accumulation of H_2_O_2_ and O_2_^−^, and enhances rice resistance to sheath blight (Tiwari et al. 2020). Various MAPKs play important roles in plant adaptive responses to biotic stresses. Silencing of *OsMAPK20-5* remarkably reduces resistance of rice to *M. oryzae*, but enhances resistance to *R. solani* (Liu et al. 2019). Another group of small, highly basic, cysteine-rich antimicrobial peptides called defensins from various plant species exhibit strong antimicrobial activities. Expression of such proteins as Ace-AMP1 from *Allium cepa*, Dm-AMP1 from *Dahlia merckii* and Rs-AFP2 from *Raphanus sativus* in rice greatly suppresses the growth of *M. oryzae* and *R. solani* (Jha et al. 2009; Jha and Chattoo 2010; Patkar and Chattoo 2006). ASR2 (short for abscisic acid, stress and ripening 2) specifically binds to an important *cis*-element GT-1 and activates the expression of the rice defense-related gene *Os2H16*. Over-expression of the stress-responsive gene *OsASR2* enhances resistance against *Xanthomonas oryzae* pv. *oryzae* and *R. solani*, and tolerance to drought in rice (Li et al. 2018). Collectively, many defense-associated genes can be manipulated to improve sheath blight resistance in rice (Table 2).

### QTL for Disease Resistance to RSB

It is well recognized that rice resistance to sheath blight is a quantitative trait controlled by multiple genes (Zuo et al. 2014a). Therefore, identification, mapping and subsequent characterization of RSB resistance QTL will be of great significance for sheath blight resistance breeding in rice (Jia et al. 2012; Molla et al. 2020; Taguchi-Shiobara et al. 2013; Yadav et al. 2015).

Since the first RSB resistance QTL was identified in 1995, more than 110 RSB resistance QTLs have been mapped to different chromosomes in rice (Molla et al. 2020; Wen et al. 2015; Zhang et al. 2019a). However, only *qSBR9-2*, *qSBR*^*11 − 1*^, *qSB-9*^*TQ*^ and *qSB-11*^*LE*^ have been finely mapped and no RSB resistance QTL has yet been isolated in rice (Channamallikarjuna et al. 2010; Zuo et al. 2013; Zuo et al. 2014a). A total of 14, 12, 12 and 26 putative genes have been predicted in the *qShB*^*9-2*^, *qSB-9*^*TQ*^, *qSB-11*^*LE*^ and *qSBR*^*11-1*^ regions, respectively (Channamallikarjuna et al. 2010; Zhang et al. 2019a; Zuo et al. 2013; Zuo et al. 2014b). The *SBR-9* locus in a 12.8-Mbp region between the markers Nag08KK18184 and Nag08KK18871 contains at least two putative resistance genes (Liu et al. 2009; Taguchi-Shiobara et al. 2013). In addition, a novel chitinase gene *LOC_Os11g47510* in the QTL *qSBR*^*11-1*^ region of the RSB resistant rice cultivar Tetep has been functionally verified. Expression of the chitinase gene in the RSB susceptible rice line significantly inhibits the growth and branching of *R. solani* hyphae and enhances rice resistance to RSB (Richa et al. 2017). A major-effect QTL for durable resistance containing a cluster of 12 germin-like protein (OsGLP) genes has been identified on chromosome 8 in rice. The more the *OsGLP* genes in this gene cluster are silenced by RNAi, the more the transgenic lines are susceptible to *R. solani*. The disease resistance conferred by OsGLPs at the QTL region, which is highly conserved in the grass family, is broad-spectrum (Manosalva et al. 2009). More importantly, an F-box protein ZmFBL41 has recently been identified to confer resistance to banded leaf and sheath blight in maize through a genome-wide association study (Li et al. 2019b). The isolation of the maize sheath blight resistance gene offers a promising avenue to generate sheath blight resistant cultivars in different crop plants.

Multiple QTLs for RSB resistance have been detected in several resistant varieties, including Teqing, Jasmine 85, Zhaiyeqing 8, Xiangzaoxian 19, Tetep and Pecos (Datta et al. 2001). Some major QTLs for RSB resistance, such as *qSB-9*^*TQ*^, *qSB-11*^*LE*^ and *qSB-11*^*HJX*^, have been utilized in resistance breeding program. Pyramiding disease resistance QTLs has been considered as an important strategy to develop RSB resistant cultivars. The lines carrying *qSB-11*^*LE*^ and *qSB-11*^*HJX*^ have a significantly lower disease level than the recurrent parent and the lines with single QTL under the same genetic background, indicating that QTL pyramiding can further increase the resistance to sheath blight (Li et al. 2019a). It has been also demonstrated that pyramiding of two QTLs (*qSB*^*9-2*^ and *qSB*^*12-1*^) and three QTLs (*qSB7*^*TQ*^, *qSB9*^*TQ*^ and *qSB11*^*LE*^) has higher levels of resistance than a single QTL (Wang et al. 2012; Yin et al. 2008). Therefore, simultaneous introduction of multiple resistance alleles and stacking of multiple QTLs in susceptible varieties increase rice resistance to RSB (Chen et al. 2014; Pinson et al. 2008; Zuo et al. 2008).

Sheath blight resistance QTLs are often mapped to the same region as the QTLs for heading date and plant height (Pinson et al. 2005; Sharma et al. 2009). For instance, four QTLs for RSB resistance have been identified in F_8_ recombinant inbred lines of two rice varieties from the United States, which are allelic to the QTLs for plant height and heading date (Goad et al. 2020). The flag leaf angle, length and plant compactness are also tightly linked with the RSB resistance QTLs, *qRlh11*, *qSBR11-3*, *qSBR11-1*, *qSBR9-1*, *qShB3-2* and *qSB-9* (Hossain et al. 2016). The reason for the correlation between morphological traits and resistance may be related to canopy microenvironment. Rice canopy density has a great impact on humidity and temperature, which are the important environment factors for pathogen infection and multiplication.

## Conclusions

Sheath blight has become one of the most important diseases in rice. Genetic resistance has been well considered as the most economic, effective and environmentally friendly strategy to control crop diseases. However, no RSB resistance gene has been isolated and identified. Increasing knowledge on molecular interaction between *R. solani* and host plants reveals many pathogenicity factors in *R. solani* and defense-associated genes in rice. Accordingly, the effective strategies to develop RSB resistant germplasms include disarming essential pathogenicity factors in *R. solani* via host-derived RNAi and transgenic technology, manipulating expression of plant defense-associated genes, and pyramiding the RSB resistance QTLs.

## Data Availability

Not applicable.
